# B Cells as a Therapeutic Target in Paediatric Rheumatic Disease

**DOI:** 10.3389/fimmu.2019.00214

**Published:** 2019-02-14

**Authors:** Meredyth G. Ll Wilkinson, Elizabeth C. Rosser

**Affiliations:** ^1^Infection, Immunity, Inflammation Programme, UCL Great Ormond Street Institute of Child Health, London, United Kingdom; ^2^Arthritis Research UK Centre for Adolescent Rheumatology, University College London, UCLH and GOSH, London, United Kingdom; ^3^NIHR Biomedical Research Centre, Great Ormond Street Hospital, London, United Kingdom

**Keywords:** B cells, autoimmunity, pediatric autoimmune diseases, inflammation, biologics

## Abstract

B cells carry out a central role in the pathogenesis of autoimmune disease. In addition to the production of autoantibodies, B cells can contribute to disease development by presenting autoantigens to autoreactive T cells and by secreting pro-inflammatory cytokines and chemokines which leads to the amplification of the inflammatory response. Targeting both the antibody-dependent and antibody-independent function of B cells in adult rheumatic disease has led to the advent of B cell targeted therapies in clinical practice. To date, whether B cell depletion could also be utilized for the treatment of pediatric disease is relatively under explored. In this review, we will discuss the role of B cells in the pathogenesis of the pediatric rheumatic diseases Juvenile Idiopathic Arthritis (JIA), Juvenile Systemic Lupus Erythematosus (JSLE) and Juvenile Dermatomyositis (JDM). We will also explore the rationale behind the use of B cell-targeted therapies in pediatric rheumatic disease by highlighting new case studies that points to their efficacy in JIA, JSLE, and JDM.

## Introduction—B Cells

The most well-defined function of B cells is their ability to produce antibodies as part of the humoral immune response. However, other described roles of B cells in the immune system have recently emerged including the ability to present both peptide and lipid antigens and to produce an array of pro and anti-inflammatory cytokines (1–3). Following development in the bone marrow where they undergo processes that aim to ensure tolerance, B cells are released into the periphery as antigen-inexperienced cells. Upon activation and exposure to antigen, B cells proliferate and differentiate into antibody-producing plasma cells or memory B cells, or, depending upon the cytokine environment in which they find themselves, into cytokine-producing B effector (Be) cells (For detailed information see Box 1). Naïve B cells differentiate into Be-1, which produce type 1 cytokines, or Be-2 cells, which produce type 2 cytokines, when co-cultured with polarized T helper (Th)-1 or Th-2 cells, respectively (4). B cells can also produce immunoregulatory cytokines such as IL-10, IL-35, and TGFβ (5, 6). Production of immunoregulatory cytokines by B cells is usually attributed to a specialized subset of B cells known as regulatory B cells which directly influence T cell function in humans by suppressing the differentiation of Th-1/Th-17 cells and inducing the differentiation of T regulatory cells (Tregs) (5). B cell activation also leads to the upregulation of molecules that mediate antigen-presentation, such as MHC class II (MHC class II), or co-stimulatory molecules such as CD86/CD80. In some B cells, activation induces class-switching, the process by which B cells change their immunoglobulin class from one to another (i.e., IgM to IgG/IgA/IgE). All together, these diverse functions within the adaptive immune response make B cells an important target for investigation in rheumatic disease.

Box 1 **Life Cycle of a B Cell**

The earliest stages of B cell development occur in the bone marrow, which provides a specialized environment containing non-lymphoid cells such as osteolineage cells and stromal cells. These cells support haematopoiesis and B cell development by secreting specific cytokines and growth factors such as IL-7, FLT3 ligand, stem cell factor (SCF), RANKL and CXCL12 (12). In this specialized environment, repression of FLT3 in haematopoietic stem cells and induction of the master transcription factor PAX5 commits these pluripotent cells to the B cell lineage as they differentiate into as pre-pro B cells. Movement through the early stages of B cell development are defined by the re-arrangement of immunoglobulin (Ig) genes, which ultimately leads to the expression of a functional antigen-specific B cell receptor (BCR). Briefly, Ig re-arrangement is initiated at the pro-B cell stage where the RAG-1/RAG-2 complex mediates recombination by inducing double strand breaks at recombination signal sequences flanking the variable (V), diversity (D), and joining (J) regions of the heavy chain locus. RAG enzymes and accessory molecules then coordinate the rejoining of V, D, and J segments, excising intervening DNA to give a single coding sequence that is ligated to the μ heavy chain locus. In frame rearrangements that allow surface expression of a functional heavy chain in association with λ5 and V preB (the pre-B cell receptor) result in cessation of heavy chain rearrangement and initiation of analogous Igκ rearrangement (13). Failure to produce a functional Igκ light chain following re-arrangement of the two Igκ light chain alleles leads to re-arrangement of the alleles within the Igλ light chain locus giving a B cell four attempts to produce a functional BCR (14). It is at this stage that the BCR is tested for autoreactivity, immature B cell undergo processes that ensue central tolerance by one of three processes: 1, receptor editing—immature B cells that react with low to high avidity self-antigens can undergo receptor editing by a secondary rearrangement at the Igκ or rearrangement of the Igλ allele; 2, Clonal anergy—immature B cells that react with low avidity self-antigen can migrate to the spleen as anergic B cells; 3, clonal deletion—immature B cells are deleted by apoptosis, which occurs at a low rate for those cells that fail receptor editing (15). It is important to note that much of what we know about the early life cycle of a B cell is derived from mouse studies and although informative there may be some differences, as yet undiscovered, in humans.

**Figure 1:**
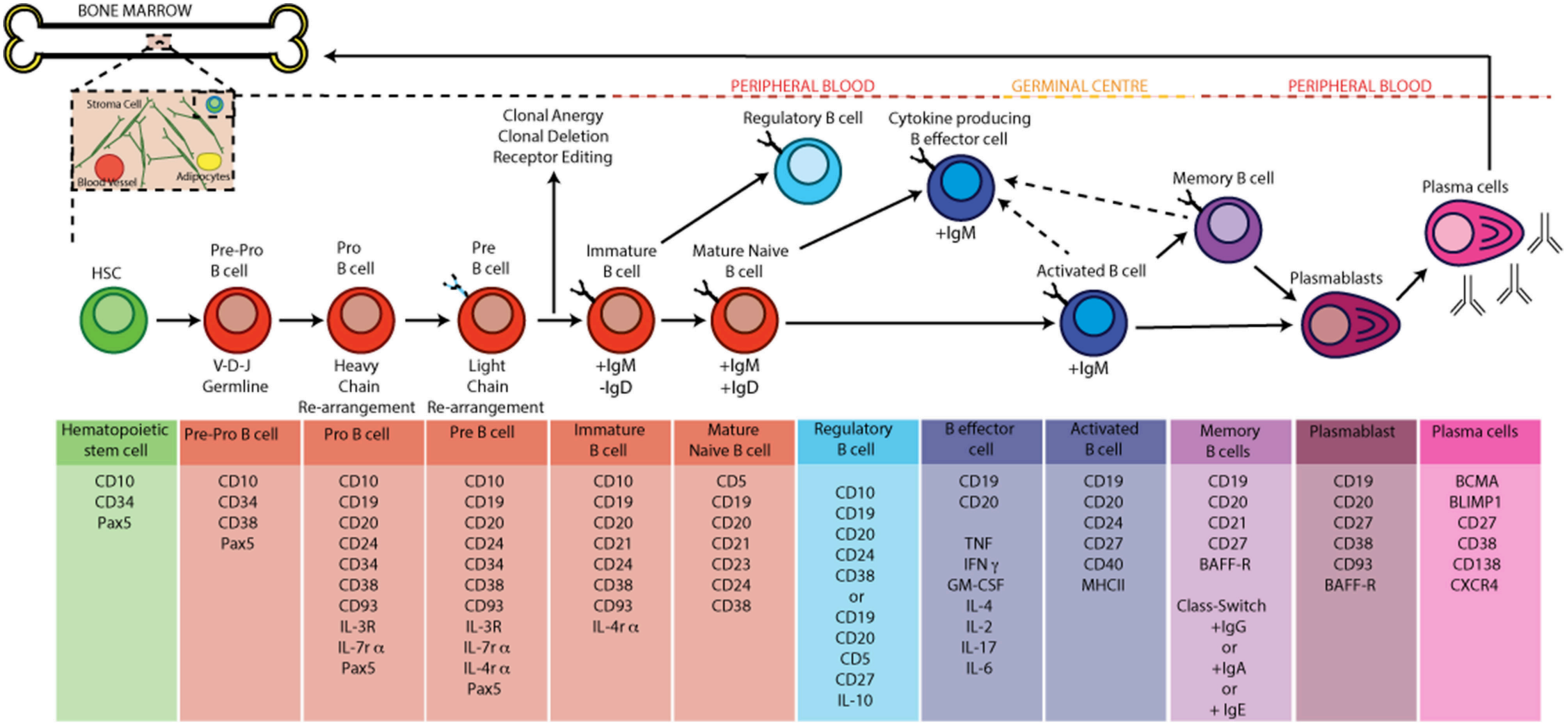
B cell development and activation in humans. B cells develop from hematopoietic stem cells generated in the bone marrow. Pro-B cells express a unique antibody, the immunoglobulin (Ig) variable domain (Fv). They undergo re-arrangement of the heavy and light chains of this immunoglobulin (V-D-J and V-J recombination) leading to the development of pre-B cells. A pre-B cell acquires antigen specificity by the expression of a unique BCR and surface IgM as it matures into an immature B cell; at the this point the B cell under goes central tolerance by one of three processes: 1, receptor editing—immature B cells that react with low to high avidity self-antigens undergo receptor editing by a secondary rearrangement at the Igκ or rearrangement of the Igλ allele; 2, Clonal anergy—immature B cells that react with low avidity self-antigen can migrate to the spleen as anergic B cells; 3—clonal deletion: occurs at a low rate for those cells that fail receptor editing. If the immature B cell survives central tolerance they develop into mature antigen-inexperienced naïve B cells. In the germinal centre B cells mature into memory and plasma cells after antigen engagement. The GC produces memory and high affinity antibody-producing plasma B cells as a result of somatic hyper mutation, clonal expansion and class switch recombination (CSR). Terminally differentiated plasma cells migrate back to the bone marrow and memory B cells produce antibody when they encounter a secondary antigen.

Once released from the bone marrow in humans, B cell subsets found in human peripheral blood can be broadly defined based on the expression of CD19, CD20, CD24, CD38, IgD, and CD27. These peripheral subsets include antigen-inexperienced immature B cells (CD20^+^CD19^+^CD24^+^CD38^+^ IgD^+^) and mature naïve B cells (CD20^+^CD19^+^CD24^int^CD38^int^ IgD^+^) as well as antigen-experienced memory B cells (CD20^+^CD19^+^CD24^+^CD38^−^ IgD^−^), plasmablasts (CD20^+^CD19^+^CD24^−^CD38^+^IgD^−^) and antibody-producing plasma cells (CD20^−^CD19^−^CD24^−^CD38^+^IgD^−^) (16). Following activation with TLR-agonists, anti-CD40 (which models T-B cell interactions *in vitro*) or pro-inflammatory cytokines (e.g., IFNα), antigen-inexperienced B cells can differentiate into IL-10 producing regulatory B cells or depending upon the cytokine-polarizing environment differentiate into cytokine producing B effector cells (5, 17). Following activation and encounter with antigen, activated B cells can also seed germinal centers (GC). Within a GC, the introduction of point mutations into the V regions B cell receptor genes by activation-induced cytidine deaminase (AICD), a process called somatic hypermutation, and subsequent competition for antigen and survival signals from T-follicular helper cells (T_FH_) by daughter B cells, a process called affinity maturation, promotes the expansion of B cells with high affinity ag-specific BCRs. Class switching of BCRs to different isotypes, guided by the nature of the immune response etc can also occur in GCs (18). Following maturation in a GC, terminally differentiated plasma cells can migrate back to the bone marrow where they can reside for up to the lifetime of the host (19, 20).The life cycle of a B cell and the markers used to identify the various stages of B cell development by flow cytometry are summarized in Figure 1.

Murine models have now established that both antibody-dependent and antibody-independent functions of B cells contribute to development of experimental models of autoimmune disease. Seminal examples include the observation that B cell-deficient mice do not develop collagen-induced arthritis (7), that CD80/CD86 expression on B cells is required for autoreactive T cell activation and the development of arthritis (8), and that chimeric mice lacking IL-10 expressing B cells develop an exacerbated arthritis that is driven by an expanded Th17 compartment (9). These data have now been translated into human disease (10, 11). However, to date, the majority of studies have focused on the role of B cell pathology in adult rheumatic disease. Although these data have underpinned the rationale for the advent of B cell depletion therapy in adult-onset disease, there is comparatively a scarcity of studies investigating whether a similar rationale could be applied to pediatric disease. In this review, we will provide a broad overview of the current evidence that B cell abnormalities drive aspects of pathology in the most common pediatric inflammatory rheumatic diseases. We will also summarize current case studies and clinical trials demonstrating that targeting B cells in these diseases could be invaluable, especially in patients whose disease is refractory to first-line therapies.

## Juvenile Idiopathic Arthritis

Juvenile Idiopathic Arthritis (JIA) is an umbrella term that encompasses a heterogenous group of conditions with a wide spectrum of outcomes. The most common rheumatic disease in childhood, JIA is characterized by joint swelling that lasts for longer than 6 weeks and that develops before the age of 16, and can cause significant disability and loss of quality of life if not controlled (21). Within this group several subtypes of JIA exist, including oligoarticular (oligo-JIA), polyarticular (poly-JIA), enthesitis-related arthritis (ERA), psoriatic arthritis (PsA), and systemic JIA (21), as defined by the International League of Associations for Rheumatology (ILAR). At present there is no cure for JIA, which means that current clinical strategy focuses on achieving clinical remission (defined as no joint inflammation and no secondary characteristics of disease such as rash, fever, serositis and uveitis (22, 23). In the absence of clinical remission, treatment is quickly escalated from the use of non-steroidal anti-inflammatory drugs to include intra-articular steroids, disease modifying anti-rheumatic drugs such as methotrexate, and in the case of methotrexate failure to expensive biologic therapies (24). The first-line biologic used in JIA treatment is TNF inhibition, and despite being well-tolerated in many children, there remains a subset of children whose disease remain uncontrolled (25, 26). At present, therapeutic strategy is defined by failure of medication in controlling disease severity as there are no clinical or biological tools that allow stratification of patients before treatment is started. Greater understanding concerning the biological pathways underlying disease development will improve stratification of patients and increase early-remission rates.

Apart from systemic JIA, which is considered to be an autoinflammatory disorder whose pathogenesis is thought to be associated with IL-6 dysregulation and macrophage activation (and will therefore not be discussed further in this review) (21), the other subtypes of JIA are classically thought of as autoimmune, T cell driven diseases. A particular role for IL-17 producing T helper cells (Th17) has been postulated, which may be particularly dominant in the extended oligo-JIA and ERA forms of JIA (27, 28). However, subtypes of JIA can also be characterized by different patterns of auto-antibody production, which implicates a central role for B cells in JIA pathogenesis. Further to autoantibody production such as anti-nuclear antibodies (ANAs), B-cells may contribute to disease pathogenesis by producing pro-inflammatory cytokines and by presenting auto-antigens to T-cells (29).

### Autoantibodies

Anti-nuclear antibodies (ANA) are most commonly detected in both oligo-JIA and rheumatoid factor (RF) negative poly-JIA suggesting a B cell involvement in both patient groups. Although the exact targets of these ANA are yet to be defined, the presence of ANA provides strong evidence that B cell tolerance is altered in both oligo-JIA and RF- poly-JIA. Indeed, it is thought that ANA+ oligo-JIA and RF- poly-JIA patients would be better considered as one subtype of disease based on similarities in pathogenesis, risk for uveitis and early disease-onset (30). ANA+ oligo-JIA and RF- poly-JIA are confined to childhood and there is thought to be no equivalent in post-pubertal patients (31, 32). While the contribution of ANA to disease pathology remains unclear, recent research has suggested that ANA positivity is associated with the development of ectopic lymphoid tissue in certain JIA patients, which by facilitating interactions between autoreactive T and B cells could directly support the production of these autoantibodies (33). In contrast to RF- poly-JIA, RF+ poly-JIA has a later onset, and many clinical and genetic features (34) that are analogous with adult-onset rheumatoid arthritis (RA), including the development of anti-citrullinated (anti-CCP) and RF autoantibodies (35). Furthermore, the multinational JIA consortium for Immunochip (JACI) recently established that RF+ poly-JIA is more genetically similar to adult-onset RA than to oligo-JIA and RF- poly-JIA (34). This provides further evidence that these diseases are likely to have similar underlying pathological mechanisms and that current pharmacological strategies employed in RA are directly relevant to RF+ poly-JIA. Although the detection of RF is used diagnostically to subcategorize poly-JIA in RF- and RF+ patients, anti-CCP antibodies are not standardly measured in clinical practice in pediatric patients despite evidence from RA patients suggesting that they may be useful in defining patients with more severe clinical disease (36, 37). An autoantibody involvement, and therefore B cell component, in the development of ERA and PsA is much less well-defined, similarly to their adult-onset counterparts Ankylosing Spondylitis and Psoriatic Arthritis, respectively. Therefore, whether B cell depletion therapy would convey much in the way of benefit for these diseases is yet to be elucidated and as such are not routinely tested.

The underlying mechanisms that lead to auto-antibody production in JIA are yet to be elucidated. Nevertheless, current data suggests that altered peripheral B cell homeostasis could be one contributing factor. CD5+ B cells, a potential human equivalent of murine B-1a cells, are expanded in the peripheral blood of patients with oligo-JIA and poly-JIA (38); B-1 cells have polyreactive B cell receptors (BCR) that recognize conserved sequences on bacterial pathogens and are therefore enriched for autoreactive epitopes. CD5+ B cells in humans are not as well-defined as there murine counterparts and may also represent a population of pre-naïve B cells (39) or activated B cells (40). There is also a reported expansion in CD24^hi^CD38^hi^ transitional B cells (41), a high proportion of which still express polyreactive BCRs, as they are still undergoing negative selection (42). These data suggest that B cell central tolerance is abnormal in JIA, leading to autoreactive B cells escaping negative selection to join the mature B cell pool. This hypothesis is supported by data that critical checkpoints in B cell tolerance are altered in JIA. For example, B cells from JIA patients are still able to undergo receptor revision in the periphery (43), a process usually restricted to the bone marrow, which is accompanied by skewed lamba:kappa light chain usage (44). Taken together, these data demonstrate that changes in the frequency of potentially autoreactive B cell subsets is accompanied with changes in the molecular events that control B cell tolerance in JIA. Future studies are needed to understand whether this directly contributes to the production of autoantibodies and how this effects JIA pathology in general.

### Cytokine Production and Antigen-Presentation by B Cells

As described above, B cells can also act as effectors of immune response by presenting antigens and producing cytokines and significantly, the CD24^hi^CD38^hi^ transitional B cell compartment which is altered in JIA also contains regulatory B cells; CD24^hi^CD38^hi^ transitional B cells produce the highest amount of IL-10 following culture with agonistic anti-CD40 or CpG (10, 45). A recent study has reported that IL-10 production by CD19^+^CD24^hi^CD38^hi^ B cells is reduced in a small cohort of JIA patients (46). There was a drastic reduction in IL-10^+^CD19^+^CD24^hi^CD38^hi^ in peripheral blood compared to controls, with an even greater reduction in synovial fluid (46). Interestingly, an initial analysis of the difference between RF+ and RF- JIA patients revealed that frequency of IL-10+CD19^+^CD24^hi^CD38^hi^ was lower in RF+ patients. It has been previously published that CD19^+^CD24^hi^CD38^hi^ Bregs are drastically reduced in RA patients, and can no longer suppress Th17 induction (11). These results strongly suggest that similarly to their adult counterparts, that in RF+ JIA patients T cell abnormalities may be at least in part driven by a dysfunctional regulatory B cell compartment. Future functional studies are needed to confirm this hypothesis.

In conjunction with a paucity of CD19^+^CD24^hi^CD38^hi^ in the synovial fluid of JIA patients (41), there is an expansion of switched memory B cells in the synovial fluid B cell compartment compared to the periphery. These switched memory B cells are phenotypically defined as CD19^+^CD27^+^IgD^−^ (41) and express high levels of the transcript for IL-12p40. Importantly, switched memory B cells in the SF express higher levels of the co-stimulatory molecules such as CD86 indicating an important role in auto-antigen presentation by these cells at the inflamed site (47). Indeed, B cells isolated from the synovial fluid of patients with JIA are more efficacious at activating isolated allogenic T cells *in vitro* compared to B cells isolated from the peripheral blood (47). To date, whether switched memory B cells differentiate in the joint or are recruited from the blood is currently not known. A recent study has shown that switched memory B cells expand at an increased rate in patients with oligo-JIA and poly-JIA and that this expansion is inhibited by anti-TNFα therapy (48). Based on these data, it could be postulated that these cells are then recruited to the joint. Collectively, evidence demonstrating that B cell abnormalities in JIA can be found both in the periphery and at the inflamed site make B cells an interesting target for therapy, particularly those patients whose disease is refractory to current treatment protocols namely non-responders to methotrexate and anti-TNFα therapy.

## Juvenile Systemic Lupus Erythematosus

Systemic lupus erythematous (SLE) is an autoimmune disease characterized by the generation of auto-antibodies directed against nuclear components. It can present with a wide variety of symptoms including renal, musculoskeletal and neuropsychiatric manifestations. The disease has a prevalence of 50–100/100,000 people in the USA and Europe (49). Patients who are diagnosed in childhood and adolescence make up 10–15% of this population with highest rates of diagnosis in female patients between 12 and 16 years (50). The juvenile-onset form of disease has many similarities with adult-onset SLE but there are some noteworthy differences in clinical manifestation. Juvenile SLE (JSLE) has a more severe disease course with higher rates of aggressive renal disease, increased mortality rates when adjusted for age and need a higher dose of glucocorticoids such as prednisolone (49, 51). Glucocorticoids are the backbone of JSLE therapy, with other DMARDs including hydroxychloroquine, aziothioprine, sulfasalazine, mycophenolate mofetil, and cyclophosphamide. For many young women, whose are diagnosed pre or peri-pubertal, these drugs have life-changing side-effects such as increasing the risk of osteoporosis, increasing the risk in infertility problems and changes in weight gain (52, 53). These side effects, coupled with the increased in mortality rates and severity of disease, demonstrate a clinically unmet need for therapeutics that substantially improve both quality of life and reduce mortality in pediatric patients.

### Autoantibodies

In the context of JSLE it is traditionally believed that autoantibodies are pathogenic through the deposition of immune complexes in the skin, renal glomerulus and sites of tissue injury, in addition to targeting specific localized antigens. More recently evidence suggests that autoantibodies act as immune modulators through the recognition of nucleic acid containing immune complexes that can directly induce cell signaling and new gene transcription through endosomal toll-like receptors (TLRs) (54). Thus, ANA positivity is a critical characteristic used to define the development of SLE and is observed in over 95% of cases. The importance of ANAs in adult SLE has been extensively reviewed elsewhere (55, 56) and due to the overlapping clinical spectra between pediatric and adult onset disease these studies are extremely informative. Both forms of the disease display positivity for a variety of ANAs including those directed against double stranded DNA (dsDNA) and extractable nuclear antigens (ENA) of which examples include anti-Sm/RNP and anti-SSA/SSB (also known as anti-Ro and anti-La autoantibodies) (55). There are however some observed differences in autoantibody profiles between the two diseases. It has been reported that there is a higher prevalence of anti-dsDNA, anti-Sm and anti-RNP antibodies in juvenile compared to adult SLE populations (57, 58), but that significantly less JSLE patients present with anti-SSA and anti-SSB antibodies (59). Whether these changes are caused by differences in the severity of pathology between SLE and JSLE remains unexplored.

Evidence on what causes the production of ANA in JSLE and SLE can be garnered from genome-wide association scanning (GWAS) studies. These studies have demonstrated that gene susceptibility loci identified in lupus patients, which include *PTPN22, BTK*, and *LYN*, are associated with the strength of BCR signaling (60). Importantly, transgenic, congenic, or knockout mice have demonstrated that modulation or deficiency in molecules that control the strength of BCR signaling leads to the productions of ANA and, in some cases, the development of lupus-like disease. Polymorphisms in *PTPN22*, whose role in BCR signaling is not well-defined, leads to hypo-responsiveness following BCR activation, impairing B cell central tolerance by diminishing both deletion and editing of autoreactive B cells (61). Conversely, gain of function mutations in *BTK* (bruton's tyrosine kinase), a major adaptor of the BCR signaling cascade, in transgenic mice leads to hyper-responsiveness of the BCR. This reduces the activation threshold of the BCR leading to spontaneous germinal center (GC) formation, a hallmark of lupus-like disease in mice, and ANA production due to ineffective deletion of autoreactive B cells during central tolerance. Similarly, mice with B cell specific deletion in *Lyn*, which is a negative regulator of BCR activation, also develop anti-dsDNA and anti-Sm antibodies, nephritis and spontaneous GC formation. These examples demonstrate that changes to the strength of B cell signaling impact activation, proliferation and both negative and positive selection of B cells, all which could lead to systemic autoimmunity in JSLE.

Autoimmunity in many mouse models of lupus is reversed by deletion of the toll-like receptors (TLRs) or the TLR adaptor protein *Myd88*. For example, in mice where there is a B cell specific deletion of Lyn, they no longer develop nephritis if *Myd88* is also deleted. Other significant examples include the observation that autoantibody profiles are altered in MRL/lpr mice based on the deletion of either TLR7 and TLR9. In humans, the TLR7 locus is found on the X chromosome suggesting that changes to X chromosome inactivation in immune cells may alter autoantibody profiles (62, 63). This is of interest as the predilection of lupus to develop in females over males is extremely high, over 90% of reported cases in adults are in females, and is especially high in individuals that develop JSLE after puberty. Future studies that stratify by sex and age are needed to address the role of X chromosome inactivation in autoantibody production and the onset of lupus nephritis. It is important to note that TLR-activation can drive both the terminal differentiation of plasma cells and memory cells and that many SLE autoantigens can be detected by the endosomal TLR compartment (64). Thus, a positive feedback loop may exist whereby activation of TLRs/BCR in lupus by nuclear antigens leads to the terminal differentiation of antinuclear B cells, which in turn produce ANA and cause immune complex deposition and antigenic spreading. Targeting these pathways are critical when considering the development of therapeutics in both SLE and JSLE.

### Cytokine Production and Antigen-Presentation by B Cells

The data summarized above demonstrate that B cell hyper-reactivity in JSLE may be, in part, driven by endosomal TLR-activation by nuclear-antigens. Of note, TLR-activation can drive both the terminal differentiation of B cells and induce cytokine production by B cells. In patients with active SLE, TLR9 expression is increased on total B cells *ex vivo* (65). Moreover, TLR9-activation *in vitro* leads to an altered cytokine profile compared to healthy B cells which is characterized by a reduction in TNFα, IL-10, and IL-6. This suggests that B cells may be chronically activated *in vivo* (66). Studies have also demonstrated that the regulatory B cell compartment is abnormal is SLE, with a seminal study demonstrating that there is an inability of CD24^hi^CD38^hi^ B cells to produce IL-10 in response to anti-CD40 stimulation and an inability to suppress inflammatory T cell differentiation *in vitro* (10). More recently, it has been established that exogenous cytokine production by other cells of the immune system also impacts B cell cytokine-production in lupus. Plasmacytoid dendritic cells (pDCs), which are the highest producers of IFNα in the immune system, are chronically activated during lupus leading to over-production of IFNα *in vivo*, which skews the differentiation of B cells away from regulatory B cells toward plasmablasts (45). These studies have focused on total lupus cohorts, encapsulating both pediatric and adult-onset patients. Future studies should stratify how B cell cytokine production is affected by SLE age of onset. In one study that did focus specifically on JSLE patients, it was shown that there is increased mRNA and protein expression of TLR3, TLR7, TLR9 in total peripheral blood mononuclear cells (PBMCs), mirroring effects seen on isolated B cells from adult-onset lupus patients. In this study, it was proposed that B cells sense apoptotic neutrophils via TLR-activation as a source of nuclear antigens in JSLE, as blocking Myd88*-*dependent signaling suppress IFNα production (67).

It is known that there are high serum levels of the B cell activating cytokine Blys in JSLE patients (68). Although, the cellular source of this Blys is unknown, it correlates with disease severity marking a subset of childhood-onset patients that develop a particularly severe form of disease (69). Studies in mice have shown that high-levels of Blys allow autoreactive B cells to escape deletion in the spleen and induce class switching of antibodies without the need for T cell help. To date, there is little research investigating the role of autoreactive T cells in the development of lupus. However, in transgenic mice, B cell specific deletion of MHCII, which prevents antigen-presentation by B cells, reduces glomerular nephritis and reduces IFNγ production by T cells (70). In this study, cognate TCR-Ag-MHCII interactions between B cells and T cells drives both T cell activation and further promotes B cell proliferation and differentiation. Although the importance of antigen-presentation by B cells is under-explored in SLE in humans, it has been demonstrated that pro-inflammatory cytokine productions by T cell is higher in patients with JSLE compared to age-matched controls (71). It is therefore tempting to postulate that altered T cell cytokine production is due to aberrant antigen-presentation by B cells.

## Juvenile Dermatomyositis

Juvenile dermatomyositis (JDM) is a rare disease, yet the most common form of childhood autoimmune myositis that presents with proximal muscle weakness and associated skin rash.

The mainstay treatments for JDM are prednisolone and methotrexate. Other immunosuppressive treatments currently used include mycophenolate mofetil, cyclophosphamide (72) and azathioprine. At present, there is limited evidence for biologic therapy and its efficacy in the treatment of JDM due to limited understanding of the mechanisms underlying disease pathology. In general adult and juvenile onset DM share a similar clinical and pathological phenotype. However, adult onset myositis patients have much higher associated risk of malignancy (73), a more chronic disease course and higher mortality rate. Key pathological differences between adult and juvenile onset DM are that JDM patients have increased risk of neovascularisation of capillaries, up-regulation of MHC class I on myofibers and type I interferon response (74). Investigating the functions of B cells in JDM may provide an insight into the mechanisms of the disease and lead to novel therapeutic pathways.

### Autoantibodies

**Figure 2:**
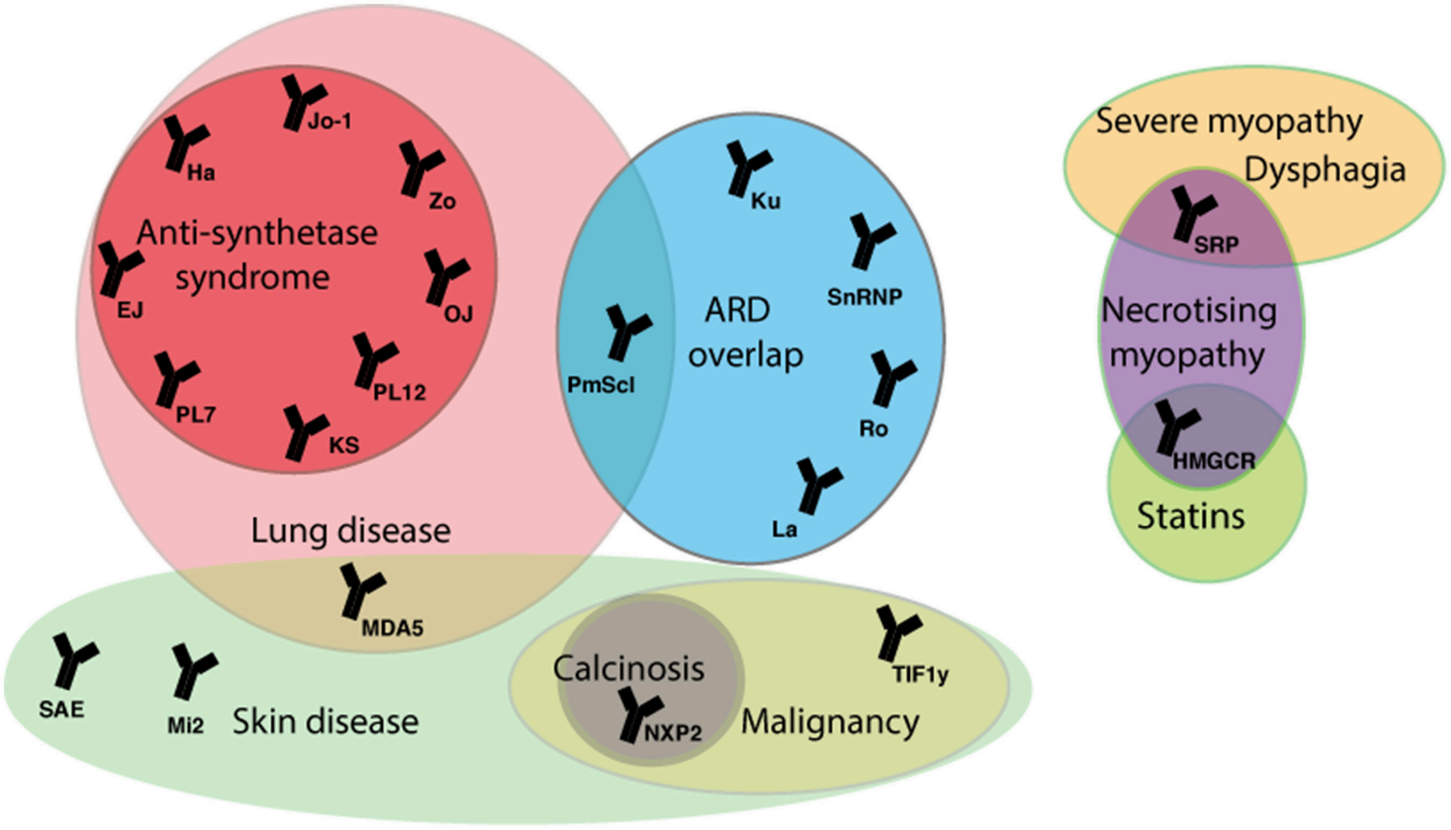
Myositis autoantibodies and their key clinical associations. JDM patients can be stratified by myositis specific and associated auto-antibodies (MSA/MAA) to group with clinical phenotype. The anti-TIF1-γ and anti-NXP-2 autoantibodies are associated with calcinosis in JDM and malignancy in adults. The MDA5 autoantibody in juvenile cases is associated with mild muscle and skin disease, but strongly associated with interstitial lung disease (ILD). In adult IIM anti-Jo-1 is the most common of the anti-synthetase autoantibodies and is associated with ILD, arthritis, fevers, Raynaud's phenomenon and mechanic's hand. The majority of mortality in adult and children IIM patients is due to ILD. In rare cases treatment with statins can trigger an immune-mediated necrotizing myopathy that can be characterized by the presence of an autoantibody against HMGCR, the pharmacological target of statins. ARD, autoimmune rheumatic disease; SRP, signal recognition particle; HMGCR, 3-hydroxy-3methylglutaryl-conenzyme A reductase; TIF1, transcription intermediary factor 1; NXP2, nuclear matrix protein 2; MDA5, melanoma differentiation-association gene 5; SAE, small ubiquitin-like modifier activating enzyme; 5NT1A, cytosolic 5′nucleotidase 1A; Mi-2, nucleosome-remodeling deacetylase complex, Jo-1, histidyl tRNA synthetase; PL7, threonyl tRNA synthetase; PL12, ananyl tRNA synthetase; OJ, isoleucyl tRNA synthetases; EJ, glycyl tRNA synthetase; KS, aspararginyl tRNA synthetase; Zo, phenylalanyl tRNA synthetase, Ha; tyrosyl tRNA synthetase; snRNP, small nuclear ribonucleic protein.

In approximately a third of JDM cases B-lymphocytes have been detected in inflamed muscle, and almost 50% of patients have detectable myositis-specific (MSA) or myositis-associated autoantibodies (MAA) (75, 76). MSA and MAA can be identified in the serum of up to 70% of JDM patients and closely correlate to specific homogenous clinical phenotypes (Figure 2). In adult myositis, the most frequent group of myositis autoantibodies detected are the anti-synthetase enzymes. The anti-synthetases are present in 25–40% of adult patients compared to 5% of juvenile patients (75). Longitudinal studies have also demonstrated that MSA phenotype and muscle biopsy score at time of diagnosis can predict the risk of staying on treatment (77). Thus, growing empirical evidence suggests that JDM patients should be stratified based on autoantibody subtype to predict both disease features, such as lung involvement or calcinosis, and to inform treatment strategies employed by clinicians (78). MSA and MAA also act as important diagnostic tools to discriminate JDM from other rheumatic diseases such as JSLE and JIA that can share some similar features of JDM, such as Raynaud's phenomenon and muscle weakness, and may lead to incorrect diagnoses (78). These antibodies are also useful to differentiate JDM from other rare immune-mediated myopathies such as those associated with anti-signal recognition particle (SRP).

The underlying cause of MSA and MAA production in JDM is yet to be elucidated. Although it has suggested that there is aberrant expression of some MAA and MSA targets, namely Jo-1 and Mi-2, on regenerating muscle fiber in myositis patients when compared to controls (79). Given the presence of these autoantigens, and that B cells have long been detected in the inflamed muscle of myositis patients (80), it may be that autoantigen specific B cell differentiation occurs in the muscle in myositis. Prospective studies that include the phenotyping of B cells recovered from inflammatory infiltrates in JDM muscle should determine whether this hypothesis is correct. Other targets of MSA and MAA antibodies include members of the nucleic acid sensing pathway (81). The connection between auto-antibodies against nucleic acid sensing molecules and the IFN signature, which is a hallmark of myositis immunopathology, is yet to be ascertained. However, taken together these data strongly suggest that aberrant sensing of nucleic acids is central in disease pathogenesis by driving both IFN production and production of autoantibodies against these molecules (82–84). Future work will need to establish what causes auto-antibody production against these molecules and what is the effect on B cell differentiation/activation.

### Cytokine Production and Antigen-Presentation by B Cells

Due to the rarity of JDM cases, it is extremely hard to carry out functional assays to understand what underlies B cell dysfunction in JDM. Nevertheless, large cohorts of JDM patients such as Juvenile Dermatomyositis Cohort Biomarker Study and Repository (UK and Ireland) (JDCBS) and the Myositis Genetics Consortium (MyoGen) are now being used to address how B cell function is altered in children with JDM (85, 86). For example, using patients recruited to the JDCBS, it was recently reported that CD19^+^CD24^hi^CD38^hi^ B cells are expanded in the peripheral blood of JDM patients that are naïve of immunosuppressive treatment, demonstrating that similarly to other rheumatic disorders normal B cell development is affected. In this study, it was also demonstrated that cytokine-production by immature B cells is altered in JDM, exhibiting a pro-inflammatory phenotype after activation through TLR7 and IFN-α (84). To our knowledge, there is only one other report showing that cytokine production is affected in JDM B cells. The data was presented as part of a larger study comparing IL-10 production by B cells from children with autoimmune disease with healthy controls. In this study, as part of a collection with JSLE and undifferentiated or overlap (mixed) connective tissue disease patients, JDM patients were shown to have a reduced percentage of B10 cells; a population of regulatory B cells that have been identified in human and mouse (87).

To date, there are no studies published that directly investigate whether antigen presentation by B cells is altered in patients with JDM. However, a GWAS study carried out by MyoGen demonstrated that susceptibility of disease was associated with SNPs within the MHC locus (88). Miller et al. have also reported that risk of JDM development was particularly associated with the HLA haplotype HLA-DRB1^*^0301 (89). Future studies are needed to directly understand how this affects auto-reactive T cell activation by B cells in JDM and whether this is altered in different autoantibody subtypes. Greater understanding of the B cell component that underlies JDM pathogenesis will provide evidence for the efficacy of B cell-targeted-therapies for this disease.

## B Cell Targeted-Therapies in PEDIATRIC Rheumatic Disease

### B Cell Depletion Therapy

The most well-known and widely used B cell depletion therapy for the treatment of rheumatic disease is Rituximab. This chimeric monoclonal antibody targets the surface protein CD20 expressed mainly by memory and naïve B cells and results in depletion by antibody-dependent cytotoxicity, complement-mediated lysis or apoptosis (90). Table 1 summarizes the B cell depletion therapeutic agents that have been trialed in pediatric rheumatological diseases. The original rationale for treatment of adult patients with rheumatoid arthritis with rituximab, circa 1998, was that depletion of the memory B cell compartment would influence immune cell interactions that could potentially “re-set” immunological tolerance as crucially auto-antibody producing plasma cells no longer express CD20 (94). Although the actual picture is more complicated, numerus randomized trials have now confirmed that B cell depletion therapy with rituximab has some beneficial effects in the treatment of adult disease.

**Table 1:** Summary of clinical trials investigating efficacy of B cell targetted therapies in pediatric rheumatic disease.

**Authors**	**Title of trial**	**Drug mechanism**	**Kind of trial Patient**	**Patient group**	**Outcome Measures**	**NICE approved**	**Summary**
Rovin et al. (91)	Lupus Nephritis Assessment with Rituximab Study (LUNAR)	Anti-lymphocyte monoclonal antibody leading to lysis of B lymphocytes.	Double-blind randomized, placebo controlled trial, Phase III	SLE (*n* = 144)	Assessed for renal response based on serum creatinine levels, urinary sediment and urine protein to creatinine ratio (UPC).	No	Despite rituximab leading to high response rate within patient cohort after the trial finished, no long-term outcomes were observed after 1 year of treatment
Merill et al., (95)	Exploratory Phase II/III SLE Evaluation of Rituximab (EXPLORER)	Anti-lymphocyte monoclonal antibody leading to lysis of B lymphocytes.	Double-blind randomized, placebo controlled trial, Phase II/III	SLE (*n* = 257)	Monthly assessments with the British Isles Lupus Assessment Group (BILAG) index and the Lupus Quality of Life (LupuQol) index, including pain and fatigue outcomes.	No	No significant differences were observed between the placebo and treatment groups
Oddis et al. (92)	Rituximab in Myositis Study (RIM Study)	Anti-lymphocyte monoclonal antibody leading to lysis of B lymphocytes.	Double-blind randomized controlled, placebo phase trial	PM (*n* = 76), DM (*n* = 76), JDM (*n* = 48)	Definition of improvement (DOI) based on International Myositis Assessment and Clinical Studies Group (IMACS). Improvement was classified as a = >20% increase in any 3 of 6 IMACS items and no more than 2 worsening items by > = 25% compared to baseline.	No	No significant differences between treatment pathways however 83% of refractory myositis patients met DOI.
Hui-Yuen et al. (93)	Pediatric Lupus Trial Of Belimumab (PLUTO)	Binds to human B lymphocyte stimulator protein (BLyS) to prevent binding on B cell receptors, interfering with B cell survival.	Observational Stud	SLE (*n* = 157), JSLE (*n* = 38)	Comparison of overall physician assessment including clinical symptoms at baseline vs. endpoint.	Yes	71% of pediatric SLE patients presented a clinical improvement within 6 months and over two thirds were able to reduce steroid use.
Curiel et al., ongoing.	Abatacept in Juvenil Dermatomyositis (AID); Assessing the safety and efficacy of subcutaneous Abatacept in refractory JDM patients.	Soluble fusion protein that inhibits T lymphocyte activity	Single group clinical trial, Phase IV	JDM (*n* = 10)	Definition of improvement (DOI) based on International Myositis Assessment and Clinical Studies Group (IMACS). Improvement was classified as a = >20% increase in any 3 of 6 IMACS items and no more than 2 worsening items by > = 25% compared to baseline.	No	Results not yet released.

Despite failing to meet primary endpoints in two large double-blind randomized, placebo-controlled trials investigating both renal [LUNAR (91)] and non-renal [EXPLORER (95)] manifestations of SLE in adults, rituximab has been subsequently demonstrated as a potentially effective treatment in both adults and children with refractory disease (96). Although not currently approved by the UK national institute of clinical excellence (NICE), rituximab can be prescribed at the discretion of the treating physician on the basis of a specialist NHS England interim commissioning policy. It has been suggested that both LUNAR and EXPLORER trials may have failed to demonstrate efficacy for a number of reasons including problems with the study design, which is frequently quoted in the cases of clinical trials investigating the efficacy of new therapeutics in SLE. Factors including high background steroid doses in the control group and issues with end-point measurements are likely to have contributed to this. It is important to note that evidence from animal models suggests that lupus-prone MRL mice are surprisingly refractory to B cell depletion due to high levels of serum IgG inhibiting FcγR-dependent phagocytosis by macrophages and neutrophils (97). Further studies are needed to understand whether lupus patients that do not respond to B cell depletion therapy have a similar acquired deficiency in their myeloid compartment caused by the burden of endogenous auto-antibody associated with disease pathology.

For pediatric rheumatic disease, very few formal trials exist that have investigated the efficiency of B cell depletion therapy in suppressing disease symptoms. In spite of this, Rituximab is increasingly being used as an adjunctive therapy to treat children with JIA, JDM and JSLE. Importantly, despite the well-established use of Rituximab in RA, it is currently infrequently used as a biologic in RF+ poly-JIA, which, as discussed above, is thought of as the early-onset equivalent of RA. Future clinical trials are essential to establish its place in the clinician's therapeutic arsenal in the treatment of RF+ poly-JIA. In terms of oligo-JIA and RF- poly-JIA, especially those that have ANA, there is considerable evidence that rituximab treatment may be a worthwhile treatment option. Several case studies have demonstrated that rituximab therapy can produce sustained clinical improvement in patients with refractory disease (98–100). Currently, rituximab is often only considered following the failure of first-line treatments such as TNF inhibitors and methotrexate in both pediatric and adult disease. Taking-into-account the strong B cell signature observed in JIA patients with early-onset disease, rituximab treatment could potentially benefit these patients if used prior to failure to first-line therapies and could therefore prevent sustained disability as a result of established joint damage in some of these patients.

In JSLE, a retrospective cohort study recently reported that the use of rituximab for the treatment of active or refractory disease is increasing. There is increasing evidence to support that this treatment is effective in reducing specific disease activity biomarkers in SLE. Rituximab has been shown to reduce steroid use (and therefore importantly decrease the incidence of side-effects associated with long term corticosteroid use) in those not responding to standard therapies (101). It has been suggested that adverse reactions to rituximab are increased in JSLE patients compared to adult-onset SLE patients' (102). For example, half the children recruited to a small study in France reported by Willems et al. developed thrombocytopenia and neutropenia following treatment. However, it is often difficult to distinguish what findings are a result of side effects to treatment with rituximab and what may be attributed to an underlying disease flare (102). In comparison, another small study by Marks et al. (103) reported no side-effects or adverse reactions. This highlights the need for larger studies to prevent sampling bias and to establish the safety profile and efficacy of rituximab in childhood onset disease. Problems with study design in adult SLE, which include the treatment of rituximab alongside many other medications and lack of an appropriate control group, are also present in JSLE. Appropriate trials in adults and children are now required as the subtle immunological differences observed between SLE and JSLE means that rituximab treatment may have a different efficacy in disease depending on age of onset.

One of the few pediatric diseases where rituximab has been trialed is in JDM, in a study by Oddis et al. where efficacy in adult DM and JDM were compared (92). Although the primary end point of the randomized control trial was not met, Oddis et al. showed that a higher proportion of JDM patients (87%) treated with rituximab met the definition of improvement more quickly than adult dermatomyositis (ADM) patients (78%). These results might imply that B cells in JDM are either more pathogenic than in ADM, or perhaps B cells have a greater regulatory role in ADM compared to JDM. Future studies are needed to address these differences.

It is important to note that some patients with pediatric rheumatic disease are refractory to first-line therapies and thus may require monoclonal antibody therapy, such as rituximab. Clinical trials that directly compare rituximab therapy with methotrexate alone, TNF inhibiting drugs or, where appropriate, anti-IL-6 therapy would be extremely informative to help stratify treatment efficacy when compared to other current treatment strategies. Development of other CD20-targeting monoclonal antibodies is ongoing, recent additions include ofatumumab (104) and veltuzumab, which have a similar mechanism of action to rituximab. Notably, ofatumumab is a fully humanized form of CD20, which has been demonstrated to be effective in adults and children with SLE who have had adverse reactions to rituximab (104).

### Other B Cell-Targeting Therapies

**Figure 3:**
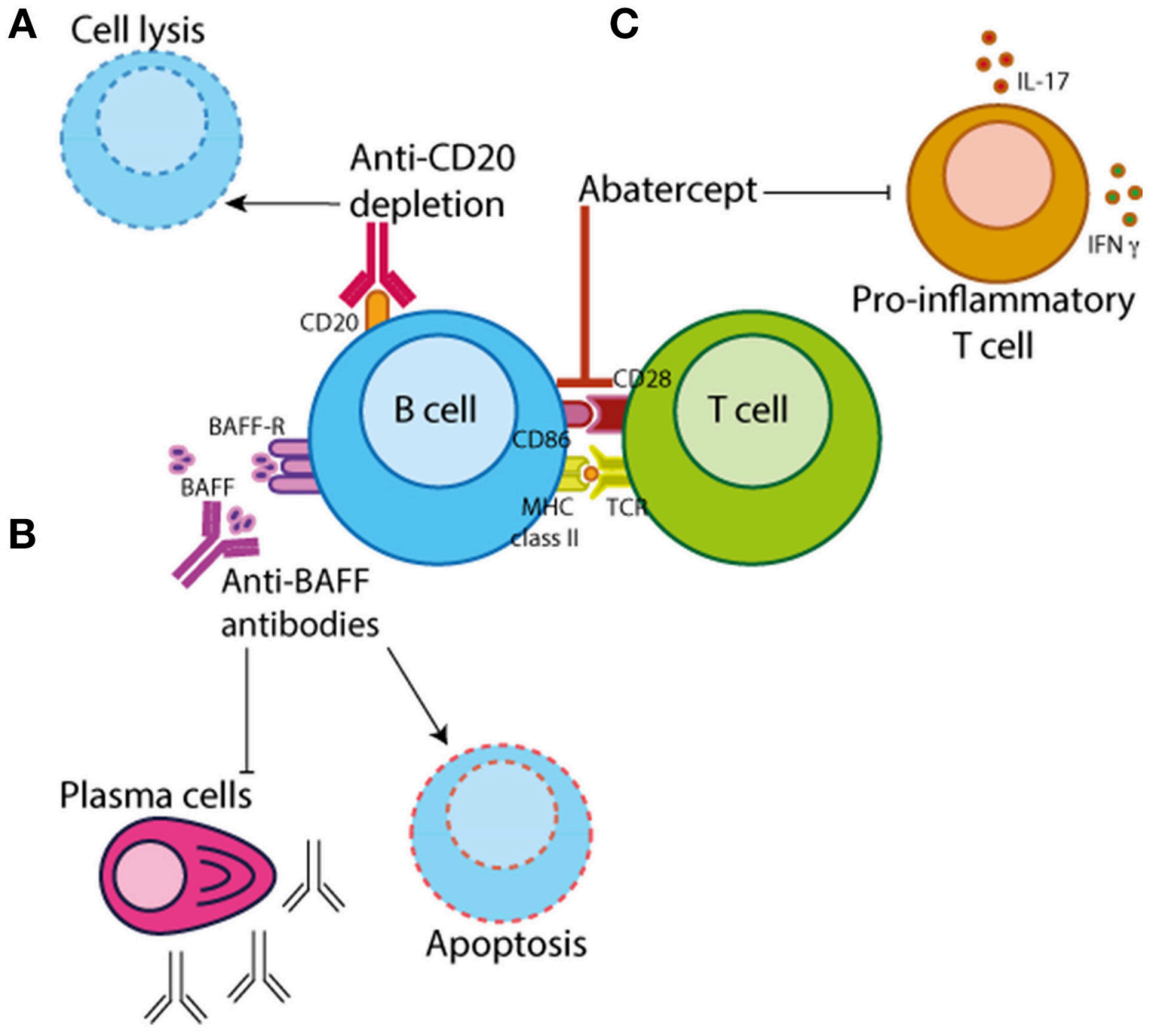
B cell-targeted therapeutics. There are three main categories of B cell targeting therapeutics. **(A)** Chimeric monoclonal antibodies against CD20 (e.g., rituximab) which target B cells for depletion by antibody-dependent cytotoxicity, complement-mediated lysis or apoptosis. **(B)** Monoclonal antibodies that bind B cell activating factor/B-lymphocyte stimulator (BAFF/BLyS) (e.g., Belimumab) preventing B cell proliferation and survival. **(C)** Abatercept, a fusion protein of CTLA-4 and a fragment of the modified Fc domain of human IgG1, that binds to CD28 on T cells preventing the interaction with CD80/CD86 on antigen-presenting cells inhibiting T cell activation and differentiation.

Further to B cell depletion therapy, there are other biologics in clinical practice with a method of action that targets B cell activation/function (Figure 3). The most well-known of which are Belimumab (a monoclonal antibody that inhibits BAFF/Blys) and Abatacept (which binds CD28 on T cells thus preventing its interaction with CD80/CD86 on antigen-presenting cells such as B cells). Data demonstrating that Blys levels in the sera differ between JIA and JSLE patients suggests that Belimumab treatment may be more efficacious in diseases that have higher levels of BAFF such as JSLE. Belimumab is currently FDA approved for the treatment of adult-onset SLE and there is currently an ongoing trial in pediatric lupus (PLUTO – NCT01649765) to investigate the safety and pharmacokinetics of this treatment in disease. Although PLUTO is yet to report, there is an abstract reporting that background steroid treatment was tapered in 63% of JSLE patients treated with Belimumab suggesting a favorable effect on disease (93). To our knowledge, similar studies do not yet exist in JIA or JDM.

Abatacept is primarily thought of as a drug that targets T cell function by preventing its interaction with antigen-presenting cells, which include both B cells and dendritic cells (DCs). Importance of B cells in the efficacy of abatacept-treatment is demonstrated by studies showing that in RA patients abatacept-treatment is dependent upon the baseline levels of memory B cells (which express higher levels of CD80/CD86) (105, 106). There is also some evidence that abatacept inhibits phosphorylation of Syk in PB B cells from RA patients, potentially directly altering intracellular signaling cascades which affect B cell activation and proliferation (107). Although, abatacept treatment is likely to affect multiple immune cell subsets, these data demonstrate a decisive effect of this drug on the B cell compartment.

At present, in the UK, NICE only recommends Abatacept treatment for poly-JIA patients who are over 6 years of age, whose disease has not responded to treatment with a disease-modifying anti-rheumatic drug (DMARD) or at least one TNF inhibitor. This means to date that no studies have directly compared the efficacy on abatacept to drugs such as adalimumab and etanercept as clinical trials have focused on patients that have failed TNF therapy (108). The safety profile of Abatacept is thought to be generally good and studies have demonstrated that there is a favorable response in 70% of patients. Studies have also suggested a possible efficacy in oligo-JIA patients with Uveitis who have also failed TNF inhibition therapy (109). In the future, it should be possible to target patients that have mutations in PTPN22, a JIA susceptibility locus, which encodes a tyrosine phosphatase that downregulates CD28 activation (110). These patients are likely to fall across all JIA subtypes.

The efficacy of Abatacept treatment for JSLE and JDM is much less well-defined. Indeed, all trials investigating its efficacy of Abatacept in adult-onset SLE have not reached their primary end-point, although this is likely to be to problems in clinical trial design in lupus, similarly to those conducted with Rituximab, as there is some evidence it suppresses the severity of nephritis (101). In JDM, there is a case study suggesting that Abatacept-treatment suppresses recalcitrant disease that is complicated by calcinosis formation and there is a phase 4 interventional clinical trial (NCT02594735) underway to assess the safety and efficacy of subcutaneous abatacept in 10 patients 7 years of age and older with refractory JDM (111). Future work is necessary to establish its efficacy in both juvenile SLE and DM patients.

## Concluding Remarks

B cell depletion therapy has improved the lives of many patients with adult-onset autoimmune rheumatic disease. The data summarized above demonstrate that B cell dysfunction is also central to the pathology of many patients with pediatric-onset rheumatic disease. However, due to inherent problems in the design and approval of clinical trials focused on pediatric-onset disease, the understanding of the efficacy of B cell depletion/targeting therapies is years behind that of adult rheumatic disease. The future of clinical practice will be centered around the stratification of patients and the increased cost-efficiency of multi-omics platforms means the age of personalized medicine is fast approaching. We propose that future stratification of JSLE, JDM, and JIA will identify a subset of patients whose disease is driven by a B cell component, which makes them ideal candidates for B cell depletion/targeting therapies, and that treatment protocols will be applied which define patients by immune-phenotype rather than by clinical manifestation. For these changes in clinical practice to take place, correctly controlled clinical trials must be performed that assess the safety, efficiency and pharmacokinetics of B cell targeting biologics in children, which is likely to be drastically different to adults. In particular, the effect of these drugs on the immature immune system should be addressed. This will provide information on particular drug profiles, but will also provide information on the processes underlying B cell development in early-life. Notably, there are relatively few studies that have carried out consensus B cell phenotyping in pre and peri-pubertal individuals. Moreover, to date, there are no studies that stratify B cell responses in children and young people by sex, despite an appreciation that there is a sex-biased development of rheumatic disease in women. Future studies are needed to improve the characterization of basic B cell biology in healthy children and those with autoimmune disease, which will aid the understanding of the usefulness of B cell depletion therapy in pediatric rheumatic disease. We believe this will allow current treatment protocols to be updated so that they more effectively target the “window-of-opportunity” based on immune-phenotype, hopefully preventing long-term disability in children with musculoskeletal disease.

## Author Contributions

ER and MW wrote the manuscript. All authors gave approval before submission.

### Conflict of Interest Statement

The authors declare that the research was conducted in the absence of any commercial or financial relationships that could be construed as a potential conflict of interest.
